# Home Monitoring of Heart Rate as a Predictor of Imminent Cardiovascular Events

**DOI:** 10.3389/fphys.2019.00341

**Published:** 2019-03-27

**Authors:** Mikko P. Tulppo, Antti M. Kiviniemi, M. Juhani Junttila, Heikki V. Huikuri

**Affiliations:** Research Unit of Internal Medicine, Medical Research Center Oulu, Oulu University Hospital, University of Oulu, Oulu, Finland

**Keywords:** heart rate, coronary artery disease, cardiovascular event, home monitoring, heart rate variability

## Abstract

**Introduction:** Previous studies have documented that day-to-day variability of heart rate (HR) has prognostic significance for cardiovascular (CV) events in general population. It is unknown how HR dynamics variate before imminent CV event in patients with coronary artery disease (CAD). Our aim was to study day-to-day variation in HR dynamics before the occurrence of CV event in patients with initially stable CAD.

**Methods:** Forty-four patients with angiographically documented CAD from ARTEMIS study measured R-R intervals on a weekly basis at home for 2 years. Home measurements were performed in controlled conditions (3 min at supine and sitting) 1–2 times per week. Eleven patients had a CV event (7 acute coronary syndromes, 1 cardiac death, 2 new onset of arrhythmia needing hospitalization and 1 stroke), which occurred 11 ± 7 months after enrolment. Mean R-R interval was analyzed prospectively from the home measurements. For the patients with new CV event, average, and standard deviation (SD) of the mean R-R interval over 8 weeks preceding the CV event were calculated. For the patients without new CV event, corresponding period was determined by the median follow-up at the occurrence of new CV event.

**Results:** There were no differences in the mean R-R interval analyzed over 8 weeks between the patients with and without new CV event. The variability of mean R-R interval over 8 weeks was greater in the patients with new CV event compared to the patients without new CV event at the supine (95 ± 34 vs. 59 ± 26 ms, *p* < 0.001) and sitting positions (92 ± 28 vs. 62 ± 24 ms, *p* < 0.001).

**Conclusion:** Day-to-day variability of mean R-R interval is greater before the new CV event in CAD patients suggesting to a more unstable cardiac autonomic regulation preceding these events.

## Introduction

Previous studies have demonstrated prognostic significance of day-to-day blood pressure and heart rate (HR) variability for cardiovascular (CV) events in general population (Kikuya et al., 2008; Johansson et al., 2012). Kikuya et al. (2008) showed that increased day-to-day variability of systolic blood pressure and HR was associated with greater CV mortality during long-term follow-up even after adjustment for traditional CV risk factors. Also Johansson et al. (2012) reported that higher variability of morning day-to-day blood pressure and HR predicted fatal and non-fatal CV events in a middle-aged general population, whereas the variability of evening day-by-day blood pressure and HR did not.

Among patient populations, increased day-to-day or visit-to-visit systolic blood pressure variability has been associated with future risk for CV events in patients with hypertension and recent transient ischemic attack or stroke (Chowdhury et al., 2014; Yu et al., 2014; Webb et al., 2018). However, the association between day-to-day variability of HR and CV events is unclear in stable coronary artery disease (CAD) patients. It is well-known that HR variability is altered among patients with ischemic heart disease compared with healthy subjects (Airaksinen et al., 1987; Huikuri et al., 1994; Bigger et al., 1995). Secondly, Adamson et al. (2004) demonstrated that HR variability, measured continuously from an implanted cardiac resynchronization device, declined a median of 16 days before the hospitalization in patients with New York Heart Association class III or IV heart failure. However, the previous studies have not reported to the role of day-to-day variation in mean HR or HR variability before imminent CV event in patients with initially stable CAD during the current treatment era. Therefore, the purpose of present study was to examine the day-to-day HR dynamics in patients with stable CAD before imminent CV event.

## Materials and Methods

### Subjects and Study Protocol

The work described here is part of a larger ARTEMIS (Innovation to Reduce Cardiovascular Complications of Diabetes at the Intersection) study taking place in the Division of Cardiology at Oulu University Hospital at 2007–2014 (Oulu, Finland). The ARTEMIS study is registered at ClinicalTrials.gov, Record 1539/31/06 identifier number NCT01426685. The study was performed according to the Declaration of Helsinki, the local research ethics committee of the Northern Ostrobothnia Hospital District approved the protocol, and all the subjects gave their written informed consent. The primary outcomes of the study have been published recently (Junttila et al., 2018).

One-hundred sixty patients with angiographically documented CAD participated a 2-years home-based exercise training intervention. This ARTEMIS sub-study is previously described elsewhere in detail (Karjalainen et al., 2015). Forty-four patients were willing to measure R-R intervals (Polar RS800; Polar Electro Oy, Kempele, Finland) weekly basis at home during follow-up. More men than women were willing to measure R-R interval at home condition. During the 2-year follow-up, patients were asked to measure R-R intervals once or twice a week immediately after waking (before breakfast and washing up) using HR monitor. The home measurement protocol started with supine position (3 min) followed by sitting position (3 min), while the patients were advised to avoid additional movements and talking. At the baseline left systolic (ejection fraction, LVEF) and diastolic function (ratio of early transmitral flow velocity to early diastolic mitral annulus velocity, E/E’) were measured with two-dimensional tissue Doppler echocardiography (Vivid 7, GE Healthcare, Wauwatosa, WI, United States). Additionally, fasting blood samples were obtained at the baseline for analysis of inflammation, lipid, and glucose metabolism.

### Analysis of R-R Interval Data

The R-R interval data were edited and analyzed in Hearts program (Heart Signal Co., Oulu, Finland). Ectopic beats and artifacts were removed from the tachogram based on visual inspection. Last 2 min for each phase was analyzed to secure stationarity of the data. Mean R-R intervals and the SD of normal-to-normal R-R intervals (SDNN) were used as time domain measures. Additionally, an autoregressive model (model 20) was performed to quantify the low- (LF, 0.04–0.15 Hz) and high-frequency (HF, 0.15–0.40 Hz) power of R-R interval oscillation from each phase.

In CAD patients with new CV event, measurements for 8 weeks preceding new CV event were included in the analyses. In patients without event, 8-weeks period corresponding to the median follow-up at the occurrence of new CV event in patients who encountered this event was included. The mean R-R interval and HR variability indexes were averaged over all recordings during the 8-weeks period within each patient. Also, the within-patient standard deviation (SD) of mean R-R interval and HR variability indexes were calculated. Finally, the mean R-R interval and HR variability indexes from the first and last two measurements of the 8-weeks period were averaged to evaluate possible trends occurring before the CV event.

### New Cardiovascular Event

The participants were contacted through a mailed questionnaire and by telephone to inquire about the possible interim hospitalization after 2 years of follow-up. The final adjudication of the reason for hospitalization was ascertained from the primary discharge diagnosis from medical records. The deaths during the 2-year follow-up were registered using emergency rescue reports, hospital and physician records, autopsy data, death certificates, and interviews with the next of kin. The cause and mode of death were reviewed and adjudicated by two independent investigators; and if needed, disagreement or uncertainty was resolved in consultation with the investigators (MJJ and HH). The CV events prospectively included cardiovascular death, resuscitation from sudden cardiac arrest, hospitalization for either heart failure, acute coronary syndrome, stroke, or arrhythmia (atrial fibrillation/flutter or ventricular tachycardia) diagnosed according to the current guidelines.

### Statistical Analyses

Shapiro–Wilk’s test was used to examine the Gaussian distribution of the data. The variables that have non-Gaussian distribution were transformed into natural logarithm before the parametric statistical tests. Independent *t*-test was used for the between-group comparisons for continuous and chi-square test for categorical variables. Significant differences in variation of the mean R-R interval and HR variability between the groups were adjusted for E/E’, use of nitrates, use of alcohol, type 2 diabetes, sex, and sex × CV event interaction. based on differences in these baseline parameters between the groups with and without new CV event, as well as significant determinants of the variation in the mean R-R interval and HR variability. Pearson correlation analyses and independent *t*-test, followed by stepwise multiple linear regression, was used to assess the main determinants of variation in the mean R-R interval and HR variability. Two-way ANOVA for repeated measurement was used to assess possible differences in the mean R-R interval and HR variability (average of first vs. last 2 measurements) between the groups with and without CV event (time, group and time × group interaction) and was adjusted for E/E’, use of nitrates, use of alcohol, type 2 diabetes, and sex. The statistical analyses were performed using SPSS software, version 22.0 (SPSS Inc., Chicago, IL, United States). A *p*-value < 0.05 was considered statistically significant.

## Results

Eleven out of 44 patients had a new CV event (7 acute coronary syndromes, 1 sudden cardiac death, 2 new onset of arrhythmia needing hospitalization, and 1 stroke) during the follow-up (event time 11 ± 7 months after enrolment). Demographic characteristics of CAD patients with and without the new CV event are presented in Table 1. CAD patients with new CV event had higher E/E’ (13 ± 4 vs. 9 ± 3, *p* = 0.005) and they were more commonly treated with nitrates than their counterparts without the CV event (55 vs. 18%, *p* = 0.028). Other characteristics did not differ statistically significantly between the groups. However, patients with new event tended to have more often type 2 diabetes and more patients were alcohol users (Table 1). The mean number of R-R interval home measurements over 8 weeks was 12 ± 3 and 12 ± 3 for patients without and with new event, respectively (*n* = ns).

**Table 1:** Baseline characteristics of coronary artery disease patients with and without a new cardiovascular event.

	New CV event	No event,	*p*-value
	*n* = 11	*n* = 33	
Men, *n*	9 (82%)	27 (82 %)	1.000
Age, year	62 5	61 6	0.873
Weight, kg	88 16	85 15	0.321
BMI, kg/m^2^	29 4	30 5	0.169
Waist, cm	103 13	100 12	0.226
Systolic BP, mmHg	139 9	134 15	0.248
Diastolic BP, mmHg	86 10	82 8	0.153
Patients with T2D, *n*	9 (82%)	18 (55%)	0.158
Exercise capacity, MET	7.0 1.9	7.4 1.9	0.593
Smoking, *n*	1 (9%)	1 (3%)	0.403
Use of alcohol, *n*	8 (73%)	15 (46%)	0.117
**History of AMI**			
NSTEMI, *n*	5 (46%)	9 (27%)	0.225
STEMI, *n*	4 (36%)	7 (21%)	0.267
**Revascularization**			
PCI, *n*	9 (82%)	20 (61%)	0.181
CABG, *n*	2 (18%)	7 (21%)	0.601
**Cardiac function**			
Resting heart rate, bpm	55 8	56 8	0.650
Maximal heart rate, bpm	127 22	137 20	0.192
LVEF, %	66 5	66 8	0.728
E/E’	13 4	9 3	0.005
**Laboratory analyses**			
HbA1c, %	5.7 0.3	5.6 0.4	0.650
Fasting plasma glucose, mmol/l	6.4 0.8	6.3 1.2	0.470
Total cholesterol, mmol/l	4.1 0.7	4.0 0.7	0.669
HDL cholesterol, mmol/l	1.1 0.2	1.3 0.3	0.171
LDL cholesterol, mmol/l	2.5 0.6	2.2 0.6	0.334
Triglycerides, mmol/l	1.8 0.8	1.4 0.9	0.052
hs-CRP, mg/l	1.3 1.5	1.7 2.8	0.574
**Medication**			
Beta blockers, *n*	10 (91%)	30 (91%)	0.699
ACEI or ARB, *n*	9 (82%)	18 (55%)	0.104
Lipids, *n*	11 (100%)	30 (91%)	0.412
Anticoagulants, *n*	11 (100%)	32 (97%)	0.750
Calcium antagonists, *n*	3 (27%)	7 (21%)	0.484
Nitrates, *n*	6 (55%)	6 (18%)	0.028
Diuretics, *n*	3 (27%)	9 (28%)	0.661


*BMI, body mass index; BP, blood pressure; T2D, type 2 diabetes; MET, metabolic equivalent; AMI, acute myocardial infarction; NSTEMI, non-ST segment elevation myocardial infarction; STEMI, ST segment elevation myocardial infarction; PCI, percutaneous coronary intervention; CABG, coronary artery by-pass grafting; LVEF, left ventricular ejection fraction; E/E’, ratio of early transmitral flow velocity to early diastolic mitral annulus velocity; HbA1c, glycated hemoglobin; HDL, high-density lipoprotein; LDL, low-density lipoprotein; hs-CRP, high-sensitivity C-reactive protein; ACEI, angiotensin conversion enzymes inhibitor; ARB, angiotensin receptor blocker.*

**FIGURE 1:**
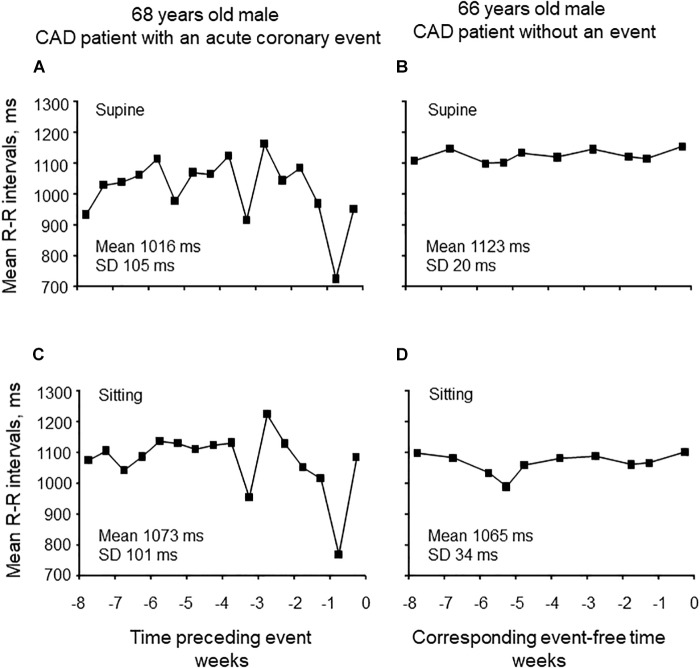
Representative examples of the variability of mean R-R intervals in supine and sitting positions for 8 weeks preceding cardiovascular event **(A,C)** and without the event **(B,D)**.

Seated or supine mean R-R interval analyzed over 8 weeks did not differ between patients without and with new CV event (Table 2). The variability of mean R-R interval was greater in patients with new CV event compared to patient without the event when measured at supine (95 ± 34 vs. 59 ± 26 ms, *p* < 0.001, and *p* = 0.006 after adjustment) and sitting position (92 ± 28 vs. 62 ± 24 ms, *p* = 0.001, and *p* < 0.001 after adjustment) (Table 2). Representative examples of the variability of mean R-R interval during 8 weeks from patients with and without CV event are shown in Figure 1.

**Table 2:** Heart rate and heart rate variability in CAD patients with and without new cardiovascular event without and with adjusted for left ventricular diastolic function and use of nitrates, alcohol, type 2 diabetes, and sex.

	New CV event	No event,	*p*-value	Adjusted
	*n* = 11	*n* = 33		*p*-value
**Supine**				
Mean R-R interval, ms	1026 112	1069 132	0.470	
SD of R-R interval, ms	95 34	59 26	<0.001	0.006
Mean SDNN, ms	43 15	44 16	0.852	
SD of SDNN, ms	12 6	14 7	0.574	
Mean HF, ln ms^2^	5.41 0.76	5.43 0.94	0.978	
SD of HF, ln ms^2^	0.74 0.23	0.56 0.23	0.024	0.003
Mean LF, ln ms^2^	5.81 1.26	5.87 0.97	0.859	
SD of LF, ln ms^2^	0.69 0.29	0.76 0.25	0.878	
**Sitting**				
Mean R-R interval, ms	983 122	1010 156	0.689	
SD of R-R interval, ms	92 28	62 24	0.001	<0.001
Mean SDNN, ms	42 15	37 13	0.259	
SD of SDNN, ms	21 18	12 5	0.105	
Mean HF, ln ms^2^	4.91 0.73	4.75 1.01	0.636	
SD of HF, ln ms^2^	0.90 0.35	0.61 0.26	0.007	0.038
Mean LF, ln ms^2^	5.53 1.18	5.53 0.85	0.994	
SD of LF, ln ms^2^	0.70 0.27	0.83 0.40	0.009	0.010


*CV, cardiovascular event; SDNN, SD of normal-to-normal R-R intervals; LF, low-frequency power of heart rate variability; HF, high-frequency power of heart rate variability.*

Any mean value of HR variability measures analyzed over 8 weeks did not differ between groups (Table 2). However, the variability of HF power over 8 weeks was greater in patients with new CV event when measured in supine (0.74 ± 0.23 vs. 0.56 ± 0.23 ms^2^, *p* = 0.012, and *p* = 0.003 after adjustment) and sitting positions (0.90 ± 0.35 vs. 0.61 ± 0.26 ms^2^, *p* = 0.007, and *p* = 0.038 after adjustment). On the contrary, the variability of LF power while seated was smaller in patients with new CV event (0.70 ± 0.27 vs. 0.83 ± 0.40 ms^2^, *p* = 0.009, and *p* = 0.010 after adjustment). The determinants of the variation in the mean R-R interval and HR variability are presented in Table 3. No significant sex × CV event interaction was observed in any HR variable.

**Table 3:** Determinants of measurement-to-measurement variation in the mean R-R interval and heart rate variability by stepwise linear regression.

	R^2^	Covariate	β	*p*-value
SD of R-R interval	0.360	CABG resting	0.418	0.001
*Supine*		HR nitrates	-0.315	0.017
			0.326	0.014
*Sitting*	–	–	–	–
SD of SDNN *Supine*	0.186	Exercise capacity	0.432	0.003
*Sitting*	0.128	Exercise capacity	0.358	0.017
SD of HF *Supine*	0.145	STEMI	0.381	0.011
*Sitting*	0.373	Smoking	0.358	0.008
		NSTEMI	-0.276	0.037
		triglycerides	0.405	0.003
SD of LF *Supine*	0.209	BMI beta	-0.347	0.017
		blockers	-0.257	0.074
*Sitting*	0.167	Smoking	0.408	0.006


*SDNN, SD of normal-to-normal R-R intervals; LF, low-frequency power of heart rate variability; HF, high-frequency power of heart rate variability, BMI, body mass index; NSTEMI, non-ST segment elevation myocardial infarction; STEMI, ST segment elevation myocardial infarction; CABG, coronary artery by-pass grafting.*

When comparing the changes in the mean R-R interval and HR variability from the first two and last two measurements during 8-weeks period, significant interaction was observed in seated and supine mean R-R interval, which increased more in the patients with new CV event compared to patients without new CV event (Table 4). Also, sitting SDNN and HF power increased more in the patients with new CV event than those without.

**Table 4:** Heart rate and heart rate variability in CAD patients with and without a new cardiovascular event as average of the two first and two last measurements of the 8-weeks period without and with adjusted for left ventricular diastolic function, use of nitrates, alcohol, type 2 diabetes, and sex.

		New CV event	No CV event,			
		*n* = 11	*n* = 33	Main effects		
				Time	Group	Interaction
**Supine**						
R-R interval, ms	*First*	976 100	1068 147	0.580	0.545	0.028
	*Last*	1052 195	1072 136			
SDNN, ms	*First*	37 17	42 16	0.829	0.814	0.816
	*Last*	45 16	46 19			
HF, ln ms^2^	*First*	4.97 1.06	5.40 0.96	0.978	0.796	0.125
	*Last*	5.47 1.13	5.42 0.93			
LF, ln ms^2^	*First*	5.52 1.48	5.80 1.04	0.746	0.911	0.931
	*Last*	5.74 1.41	6.00 0.98			
**Sitting**						
R-R interval, ms	*First*	944 142	1005 179	0.223	0.632	0.050
	*Last*	994 173	1013 170			
SDNN, ms	*First*	30 13	36 15	0.625	0.253	0.012
	*Last*	50 38	39 15			
HF, ln ms^2^	*First*	4.31 0.88	4.70 1.22	0.297	0.810	0.009
	*Last*	5.02 1.35	4.75 1.12			
LF, ln ms^2^	*First*	5.13 1.08	5.39 1.05	0.310	0.505	0.129
	*Last*	5.74 1.68	5.60 0.86			


*CV, cardiovascular event; SDNN, SD of normal-to-normal R-R intervals; LF, low-frequency power of heart rate variability; HF, high-frequency power of heart rate variability.*

## Discussion

The main finding of the present study was that day-to-day variability of mean R-R interval was greater before new CV event in patients with initially stable CAD. The analysis of day-to-day variation of HR variability measures did not provide additional information to the variation of mean R-R interval. The present results suggest that a more unstable autonomic balance precedes the new CV events. Therefore, home monitoring of resting HR may become a simple and useful method for providing prognostic information on the risk of imminent CV event in patients with stable CAD.

The risk of CV events related to increased day-to-day variability blood pressure and HR have been well documented in general populations (Kikuya et al., 2008; Johansson et al., 2012). Temporal changes HR and HR variability before imminent CV event are evidently less studied. Adamson et al. (2004) demonstrated that HR variability decreased, and the mean HR increased before hospitalization among patients with heart failure. However, daily variation in HR variability or mean HR were not reported. Furthermore, home telemonitoring, using, e.g., HR, blood pressure, and weight control, have shown to reduce hospitalization in heart failure patients (Koulaouzidis et al., 2016; Sardu et al., 2016; Tse et al., 2018). In the present study, no significant differences were found in HR variability or mean R-R interval between the patients with and without new CV event, when averaged over 8 weeks preceding the CV event or corresponding period in event-free patients. Instead, we observed that day-to-day variation in the mean R-R interval and HF power of HR variability was greater among patients who encountered the new CV event compared to those who did not. One possible explanation for the present finding is fluctuating cardiac autonomic regulation with progressing myocardial ischemia, which was underlying most of the new CV events in the present study. The triggers, such as physical, emotional and other stressors, and, thus, symptoms of angina pectoris may vary day-to-day which may well explain the present findings (Task Force Members et al., 2013). Notably, higher variation of the mean R-R interval was partly explained by the history of coronary artery bypass grafting and the use of nitrates indicating a more advanced CAD. We do not have information about patients’ compliance and symptom-based need for nitrate medication that may possibly contribute to daily variation of angina pectoris and cardiac autonomic regulation. However, the difference in the variation of the mean R-R interval and HR variability between patients with and without new CV event remained significant even after adjustments for these confounders.

We observed increase in SDNN as well as in the mean R-R interval and HF power during the 8-weeks period preceding the new CV event compared to event-free control group. This is contradictory to our expectations since acute decrease in cardiac vagal activity has been typically observed at the onset of myocardial ischemia (Sroka et al., 1997). The present findings also oppose the previous results regarding hospitalization due to heart failure by Adamson et al. (2004) one possible explanation for these divergent findings is that assessed progression of HR variability before hospitalization due to heart failure, whereas the present study assessed HR dynamics before the new CV event that were mainly related to progression new coronary events. While increased sympathetic activity and decreased vagal activity with progressing heart failure is well documented (Nolan et al., 1992; van de Borne et al., 1997), myocardial ischemia may produce varying autonomic responses. For example, Lombardi et al. (1996) showed that autonomic response to acute coronary syndrome depends on the localization of myocardial ischemia, i.e., anterior wall ischemia may induce sympathetic activation and inferior wall ischemia vagal activation. Therefore, decreased HR variability with progressing CAD may not be constantly observed. Secondly, a co-activation of vagal and sympathetic outflow may result in either increased HR variability due to the sudden and large beat-to-beat R-R interval changes or decreased HR variability due to the sudden onset of fixed R-R interval dynamics (Tulppo et al., 1998; Tulppo et al., 2005). The episodes of co-activation of both autonomic arms during short-term R-R interval recordings, such as in the present study, may partly explain our inconsistent HR variability dynamics before CV events.

The present study may have important practical implications. It seems that monitoring of the variation of mean HR may suffice to evaluate risk for imminent CV event because analysis of HR variability did not provide additional information. In addition, HR measurements at seated position appeared to be related to the occurrence of CV event more than supine measurements. These findings suggest that HR monitoring may be easily accompanied with home monitoring of BP. Some caution may be needed since HR, acquired during BP measurement, may not be as robust as mean HR obtained from R-R interval measurements. Finally, it must be acknowledged that HR dynamics may complement a symptom-based follow-up of CAD patients as it may also capture autonomic alterations also related to silent myocardial ischemia (Goseki et al., 1994).

## Limitations

The major limitation of the present study is the lack of ECG data measured at the home condition. Unfortunately, at the time of our data collection (starting 2007) we did not have suitable date recorders available to collect ECG at home. Remote technologies to measure not only ECG but also respiratory frequency, temperature, physical activity, etc., are developing very rapidly. Today, ECG and other signals can be measured at home and send data easily to the health care centers via internet (Fung et al., 2015). The present study is also limited by the small number of patients having CV event during the follow-up. Therefore, more research will be needed in a larger sample size to confirm the definite value of day-to-day variation of HR and HR variability to provide additional information of CV risk among CAD patients. Also, it must be considered that the measurements at home were short and could not be strictly controlled for potential confounders. However, they were performed in real-life conditions that may have greater value for practical implications. Additionally, stationary data can be obtained with very short stabilization period (Flatt and Esco, 2016).

## Conclusion

Day-to-day variability of mean R-R interval was greater before new CV event in CAD patients suggesting to a more unstable autonomic balance preceding this event. If confirmed in a large sample size, home HR measurement may become a simple and useful method for providing information on the risk of imminent CV event in patients with stable CAD.

## Data Availability

All datasets generated for this study are included in the manuscript and/or the supplementary files.

## Author Contributions

MT, AK, and HH contributed conception and design of the study. MT and AK organized the database and performed the statistical analysis. MT wrote the first draft of the manuscript. MT, AK, MJJ, and HH wrote the sections of the manuscript. All authors contributed to manuscript revised, read, and approved the submitted version.

## Conflict of Interest Statement

The authors declare that the research was conducted in the absence of any commercial or financial relationships that could be construed as a potential conflict of interest.
